# Comparison of rectal swabs and fecal samples for the detection of *Clostridioides difficile* infections with a new in-house PCR assay

**DOI:** 10.1128/spectrum.00225-24

**Published:** 2024-04-30

**Authors:** Ann Huletsky, Vivian G. Loo, Yves Longtin, Jean Longtin, Sylvie Trottier, Cécile L. Tremblay, Rodica Gilca, Christian Lavallée, Éliel Brochu, Ève Bérubé, Martine Bastien, Marthe Bernier, Martin Gagnon, Johanne Frenette, Julie Bestman-Smith, Louise Deschênes, Michel G. Bergeron

**Affiliations:** 1 Centre de recherche en infectiologie de l’Université Laval, Québec City, Canada; 2 Centre de recherche du Centre hospitalier universitaire de Québec-Université Laval, Québec City, Canada; 3 Axe maladies infectieuses et immunitaires, Centre de recherche du Centre hospitalier universitaire de Québec-Université Laval, Québec City, Canada; 4 Division of Infectious Diseases, Department of Medical Microbiology, McGill University Health Centre, Montréal, Canada; 5 Faculty of Medicine, McGill University, Montréal, Canada; 6 Sir Mortimer B. Davis Jewish General Hospital, Montréal, Canada; 7 Centre hospitalier universitaire de Québec-Université Laval, Québec City, Canada; 8 Centre de recherche du Centre hospitalier de l’Université de Montréal, Montréal, Canada; 9 Département de microbiologie, infectiologie et immunologie, Université de Montréal, Montréal, Canada; 10 Département de médecine sociale et préventive, Faculté de médecine, Université Laval, Québec City, Canada; 11 Département de risque biologique et de la santé au travail, Institut national de santé publique du Québec, Québec City, Canada; 12 Service de maladies infectieuses et de microbiologie, Département de médecine spécialisée, Hôpital Maisonneuve-Rosemont - CIUSSS de l'Est-de-l'Ile-de-Montréal, Montréal, Canada; 13 Département clinique de médecine de laboratoire, Centre hospitalier de l'Université de Montréal, Montréal, Canada; 14 Service de microbiologie-infectiologie, Centre hospitalier universitaire de Québec-Université Laval, Québec City, Canada; Yale School of Public Health, New Haven, Connecticut, USA

**Keywords:** *Clostridioides difficile*, diagnostics, molecular methods, rectal swabs

## Abstract

The detection of *Clostridioides difficile* infections (CDI) relies on testing the stool of patients by toxin antigen detection or PCR methods. Although PCR and antigenic methods have significantly reduced the time to results, delays in stool collection can significantly add to the turnaround time. The use of rectal swabs to detect *C. difficile* could considerably reduce the time to diagnosis of CDI. We developed a new rapid PCR assay for the detection of *C. difficile* and evaluated this PCR assay on both stool and rectal swab specimens. We recruited a total of 623 patients suspected of *C. difficile* infection. Stool samples and rectal swabs were collected from each patient and tested by our PCR assay. Stool samples were also tested by the cell cytotoxicity neutralization assay (CCNA) as a reference. The PCR assay detected *C. difficile* in 60 stool specimens and 61 rectal swabs for the 64 patients whose stool samples were positive for *C. difficile* by CCNA. The PCR assay detected an additional 35 and 36 stool and rectal swab specimens positive for *C. difficile*, respectively, for sensitivity with stools and rectal swabs of 93.8% and 95.3%, specificity of 93.7% and 93.6%, positive predictive values of 63.2% and 62.9%, and negative predictive values of 99.2% and 99.4%. Detection of *C. difficile* using PCR on stools or rectal swabs yielded reliable and similar results. The use of PCR tests on rectal swabs could reduce turnaround time for CDI detection, thus improving CDI management and control of *C. difficile* transmission.

**IMPORTANCE:**

*Clostridioides difficile* infection (CDI) is the leading cause of healthcare-associated diarrhea, resulting in high morbidity, mortality, and economic burden. In clinical laboratories, CDI testing is currently performed on stool samples collected from patients with diarrhea. However, the diagnosis of CDI can be delayed by the time required to collect stool samples. Barriers to sample collection could be overcome by using a rectal swab instead of a stool sample. Our study showed that CDI can be identified rapidly and reliably by a new PCR assay developed in our laboratory on both stool and rectal swab specimens. The use of PCR tests on rectal swabs could reduce the time for the detection of CDI and improve the management of this infection. It should also provide a useful alternative for infection-control practitioners to better control the spread of *C. difficile*.

## INTRODUCTION

*Clostridioides difficile* (formerly *Clostridium difficile*) infection (CDI) is the leading cause of healthcare-associated diarrhea, resulting in high morbidity, mortality, and economic burden (1). The clinical presentation of CDI ranges in severity from mild diarrhea to life-threatening fulminant colitis (2, 3). Patients with symptomatic CDI are considered to be responsible for most healthcare-associated transmission events, but asymptomatic *C. difficile* carriers also contribute to transmission (4–7). A rapid and accurate diagnosis of CDI is essential to guide management and prevent transmission (8, 9). CDI testing in clinical laboratories is currently performed on stool samples collected from patients with diarrhea who are analyzed for the presence of toxigenic *C. difficile* mainly by antigenic methods or PCR (10–12). However, the diagnosis of CDI can be delayed by the time required for stool collection (13). To screen for carriers of *C. difficile* and other multi-drug-resistant enteric pathogens, testing of a rectal or perirectal swab by culture or by PCR is commonly used (6, 14–20) and has been shown to provide a reliable strategy for *C. difficile* detection (21–25). The aim of the study was to evaluate the diagnostic performance of rectal swabs in comparison with stool samples to detect toxigenic *C. difficile* in patients with suspected CDI using a novel in-house PCR assay for *C. difficile*.

## MATERIALS AND METHODS

### Study design

We performed a multicenter prospective observational cross-sectional study in four hospitals that included Centre Hospitalier Universitaire (CHU) de Québec-Université Laval, McGill University Health Center (Montréal, Canada), Sir Mortimer B. Davis Jewish General Hospital (Montréal, Canada), Centre Hospitalier de l'Université de Montréal (Montréal, Canada), and Hôpital Maisonneuve-Rosemont (Montréal, Canada). Over a 28-month period (from May 2017 to August 2019), a total of 623 patients who were ≥18 years of age with ≥3 unformed stools within 24 hours, suspected of having CDI and for whom a request for *C. difficile* testing by hospital personnel was ordered in hospital wards, were enrolled in the study.

### Collection and culture of specimens

**Fig 1:**
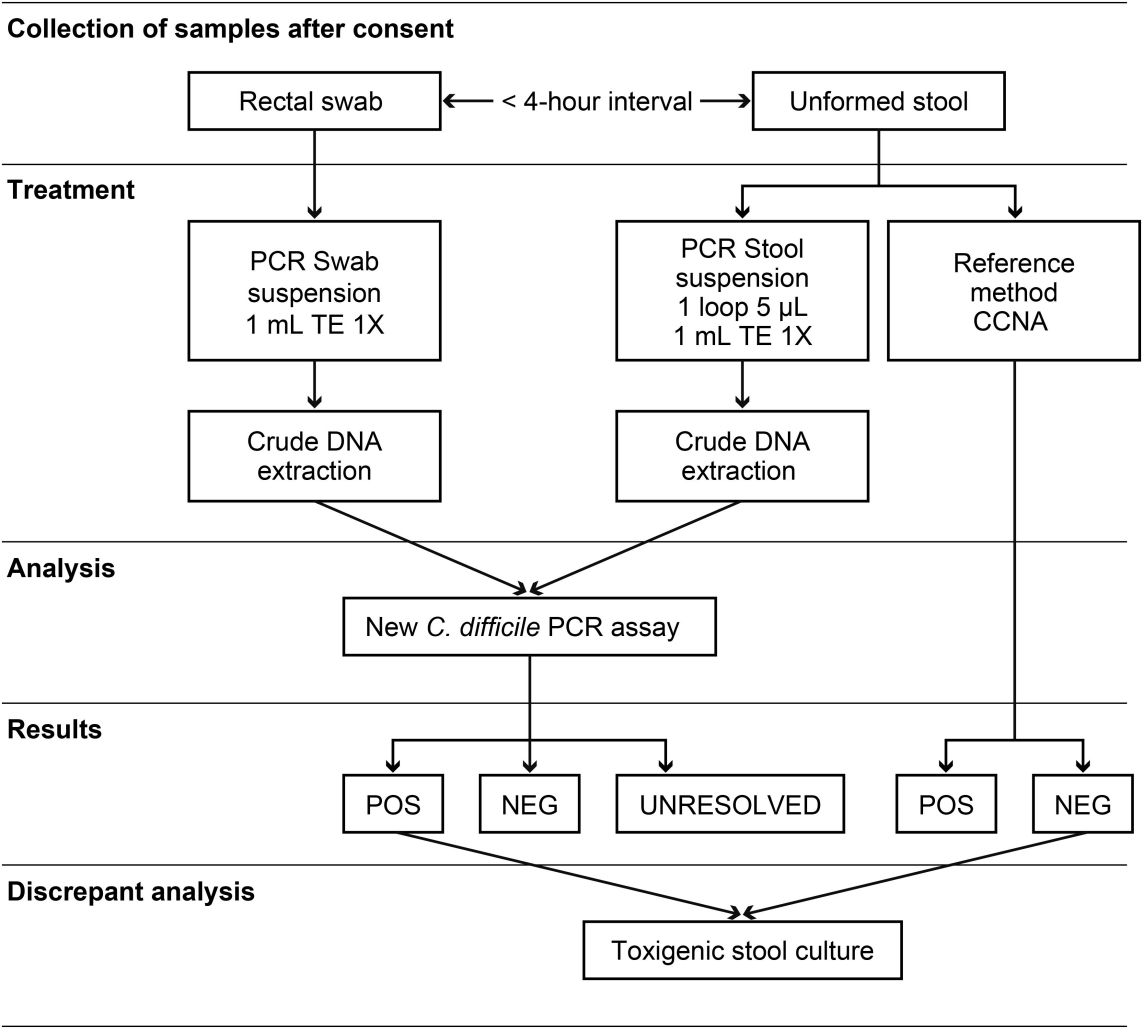
Flow chart.

For each eligible patient, a rectal swab specimen was collected by the research nurse with a collection swab (BBL Culture Swab, Liquid Stuart, Becton Dickinson) after written informed consent was obtained. Residual unformed stool, collected by the healthcare worker according to the standard hospital procedures in a dry container, was also obtained from the hospital microbiology laboratory, or unformed stool was collected by the research nurse by the same procedures. Rectal swab sample collection had to be performed within 4 hours of stool collection (Fig. 1). All specimens were kept at 4°C and used for sample preparation within 7 days after collection. The rectal swabs were tested by PCR only, and the residual unformed stool specimens were tested by both PCR and cell cytotoxin neutralization assay (CCNA) as the reference method. Stool aliquots were preserved in 15% glycerol for subsequent analysis. Data regarding medical conditions and antibiotic treatment received in the 7 days prior to stool collection were obtained from medical records.

### CCNA and toxigenic culture of stools

The residual unformed stool samples were tested by the CCNA (26) as the reference method for comparison of PCR tests performed on both rectal swabs and stool specimens (Fig. 1). The *C. difficile* toxigenic culture (TC) was performed as described previously (27) using stools frozen in 15% glycerol when it was necessary to resolve discrepant results.

### Preparation of stool samples and rectal swabs for PCR

A volume of approximately 5 µL of stool specimens was transferred into 1 mL sample buffer (SB) [(10 mM Tris pH 8.0 and 1 mM EDTA (TE)] with the use of a disposable transfer loop and kept at −80°C. Rectal swabs were immersed into 1 mL SB and kept frozen at −80°C. Frozen stool and rectal swab specimens placed into SB were thawed, and crude DNA was extracted by means of a rapid method (~10 min) previously described (28). Crude DNA extracts were stored at −80°C after PCR testing.

### PCR test

The *C. difficile*-specific PCR assay comprised in-house two forward primers, two reverse primers (Integrated DNA Technologies), and a CAL Red 610-labeled locked nucleic acid (LNA) TaqMan probe (Biosearch Technologies) targeting the *tcdB* gene (toxin B) as well as a forward primer, a reverse primer, and a FAM-labeled LNA TaqMan probe specific to internal control (IC) previously described (29). Primers and probes are described in Table S1. Amplification reactions were performed in a 21 µL reaction mixture containing 0.8 µM (each) of *C. difficile* primers, 0.4 µM of *C. difficile* probe, 0.15 µM (each) of IC primers, 0.2 µM of IC probe, 0.2 mM of dNTPs, 1 mg/mL of bovine serum albumin (Sigma-Aldrich), 4 mM of MgCl_2_ (Sigma-Aldrich), 5 U of AptaTaq Fast DNA polymerase (Roche Diagnostics), 1× AptaTaq Fast DNA polymerase buffer (Roche Diagnostics), 750 genome copies of purified nucleic acid IC, and purified genomic DNA or crude DNA extracts from the stool and rectal swab specimens for the clinical evaluation. PCR amplification (45 cycles of two steps consisting of 95°C for 4 s and 60°C for 10 s) were performed by using a CFX96 Touch thermocycler (Bio-Rad Laboratories). Negative controls contained TE buffer. Positive controls contained 10 genome copies of purified genomic DNA from toxigenic *C. difficile*. PCR was repeated using frozen crude DNA extracts when it was necessary to resolve discrepant results.

The analytical sensitivity [limit of detection (LOD)] of the PCR test was determined by testing seven different *C. difficile* genomic DNA concentrations by quintuplicate and repeating the experiment three times. *In silico* ubiquity (inclusivity) analysis of primers and probes was performed using 15,136 *C*. *difficile tcdB* gene sequences available in the National Center for Biotechnology Information (NCBI) (https://www.ncbi.nlm.nih.gov/). *In silico* analytical specificity (cross-reactivity) analysis of the *C. difficile* primers was performed against the genome sequence of the organisms listed in Table 1 using the NCBI Primer Blast tool (https://www.ncbi.nlm.nih.gov/tools/primer-blast/) with the nr database.

**TABLE 1:** List of organisms used for *in silico* specificity (cross-reactivity) analysis of the *C. difficile* primers and probes

Bacteria	
*Abiotrophia defectiva*	*Escherichia coli* O157:H7
*Acinetobacter baumannii*	*Flavonifractor plautii*
*Aeromonas hydrophila*	*Helicobacter pylori*
*Alcaligenes faecalis* subsp. *faecalis*	*Klebsiella oxytoca*
*Bacillus cereus*	*Lactobacillus acidophilus*
*Bacteroides fragilis*	*Listeria monocytogenes*
*Campylobacter coli*	*Peptostreptococcus anaerobius*
*Campylobacter jejuni*	*Plesiomonas shigelloides*
*Campylobacter jejuni* subsp. *jejuni*	*Porphyromonas asaccharolytica*
*Citrobacter freundii*	*Prevotella melaninogenica*
*Clostridium bifermentans*	*Proteus mirabilis*
*Clostridium butyricum*	*Providencia alcalifaciens*
*Clostridioides difficile* (non-toxigenic)—ATCC 43593	*Pseudomonas aeruginosa*
*Clostridioides difficile* (non-toxigenic)—ATCC 700057	*Salmonella enterica* subsp. *arizonae*
*Clostridium haemolyticum*	*Salmonella enterica* subsp. *enterica* serovar Choleraesuis
*Clostridium novyi*	*Salmonella enterica* subsp. *enterica* serovar Typhimurium
*Clostridium perfringens*	*Serratia liquefaciens*
*Clostridium scindens*	*Serratia marcescens* subsp. *marcescens*
*Clostridium septicum*	*Shigella boydii*
*Clostridium sordellii*	*Shigella dysenteriae*
*Clostridium sporogenes*	*Shigella sonnei*
*Edwardsiella tarda*	*Staphylococcus aureus* subsp. *aureus*
*Enterobacter aerogenes*	*Staphylococcus epidermidis*
*Enterobacter cloacae* subsp. *cloacae*	*Streptococcus agalactiae*
*Enterococcus faecalis*	*Vibrio parahaemolyticus*
*Escherichia coli*	
Yeast	
*Candida albicans*	
Viruses	
Adenoviridae	Cytomegalovirus
Coxsackievirus	Norovirus
Echovirus	Rotavirus
Enterovirus D68	
Human (*Homo sapiens*)	

## RESULTS

### Analytical performance of the PCR test

The LOD of the *C. difficile* PCR test was 3.6 genome copies per PCR reaction. Specificity analysis revealed no cross-reactivity of the *C. difficile* primers with species other than toxigenic *C. difficile* listed in Table 1. Ubiquity analysis showed that 15,132/15,136 (99.97%) of the *C. difficile tcdB* gene sequences presented 100% identity with the primers and the probe. One of the remaining four sequences had an unpaired base (mismatch) at the 5′ end of forward primers, which should not affect amplification efficiency, while the three other sequences had a mismatch in the probe, which could only slightly reduce sensitivity.

### Clinical performance of the PCR test

**Fig 2:**
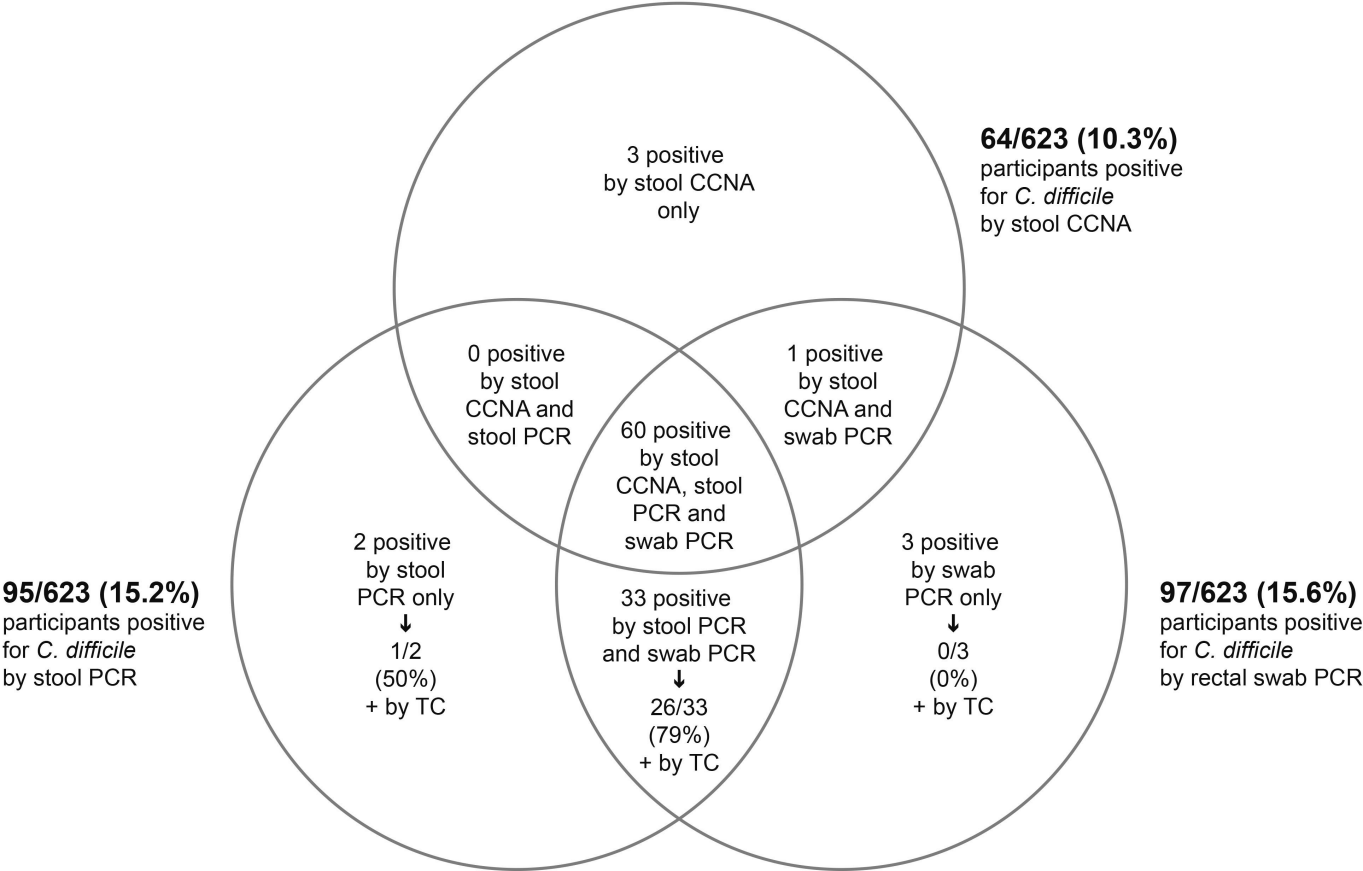
Venn diagram describing the relationship between stool and rectal swab samples positive by cell cytotoxicity neutralization assay and *C. difficile* PCR among 623 participants.

Of the 623 stool specimens obtained from patients who were tested for *C. difficile*, 64 (10.3%) were positive for *C. difficile* by CCNA (Fig. 2). Of the 64 CCNA-positive stools, 60 were PCR positive and 4 were PCR negative, while 61 rectal swabs collected from these patients were PCR positive and none were PCR negative (Table 2). The proportion of samples positive for *C. difficile* using PCR on rectal swabs or stools yielded similar results (15.2% vs 15.6%; *P* = 0.85 by McNemar Test).

**TABLE 2:** Clinical sensitivity, specificity, and predictive values of PCR for the detection of *C. difficile* from 623 rectal swabs and stool specimens

Parameter	Sample type	
	Stools	Rectal swabs
Sensitivity (95% CI)**^*a*^**	93.8% (84.8–98.3) (60/64)	95.3% (86.9–99.0) (61/64)
Specificity (95% CI)^*b*^	93.7% (91.4–95.6) (524/559)	93.6% (91.2–95.5) (523/559)
PPV (95% CI)**^*c*^**	63.2% (52.6–72.8) (60/95)	62.9% (52.5–72.5) (61/97)
NPV (95% CI)**^*d*^**	99.2% (98.1–99.8) (524/528)	99.4% (98.3–99.9) (523/526)

^*a*^ Number of both CCNA and PCR-positive results/number of CCNA-positive results.

^*b*^ Number of both CCNA and PCR-negative results/number of CCNA-negative results.

^*c*^ PPV, positive predictive value, number of both CCNA and PCR-positive results/number of PCR-positive results.

^*d*^ NPV, negative predictive value, number of both CCNA and PCR-negative results/number of PCR-negative results.

Of the 559 CCNA-negative stools, 524 were PCR negative and 35 were PCR positive, while 523 rectal swabs collected from these patients were PCR negative. Compared with the CCNA reference method, the real-time PCR assay with stool specimens had a sensitivity of 93.8%, a specificity of 93.7%, a positive predictive value (PPV) of 63.2%, and a negative predictive value (NPV) of 99.2%, while the PCR assay on rectal swabs had a sensitivity of 95.3%, a specificity of 93.6%, a PPV of 62.9%, and a negative NPV of 99.4% (Table 2). There was no PCR inhibition for all stool and rectal swab specimens tested by the PCR assay.

Additional experiments were performed to resolve the discrepancy between PCR and CCNA results. A total of 35 stool specimens and 36 rectal swabs were positive by PCR in participants with CCNA-negative stools, and 33 of these participants had PCR-positive results with both stools and rectal swabs (Table 3). All CCNA-negative stool specimens of participants for which PCR was positive with stools and/or rectal swabs were subjected to TC. Of the 35 CCNA-negative stool specimens with positive stool PCR results, 27 were shown to be positive for *C. difficile* after TC. Of the 36 CCNA-negative stool samples from participants with positive rectal swab PCR results, 26 were positive for *C. difficile* after TC (Table 3). Most participants who were stool CCNA negative but positive by both stool PCR and swab PCR were positive by stool TC (26/33, 79%).

**TABLE 3:** Retesting of specimens with discrepant results^*a*^

Results	Stools		Rectal swabs	
	First testing	Retesting	First testing	Retesting
PCR neg–ref pos/ref pos (false negative)	4/64	4/91	3/64	4/91
PCR pos–ref neg/ref neg (false positive)	35/559	8/532	36/559	10/532

^*a*^ The CCNA-negative stool specimens of participants with PCR-positive results on rectal swabs and/or stool specimens were subjected to toxigenic culture.

A total of four stool specimens and three rectal swabs were negative by PCR in participants with CCNA-positive stools (Table 3). The stool specimens of the three participants with PCR-negative rectal swabs were also PCR negative. The three PCR-negative rectal swabs remained negative after PCR re-testing, while one of the four PCR-negative stool specimens was shown to be positive after PCR re-testing with a Ct value of 37.21. The rectal swab of this participant was PCR negative.

Therefore, at the conclusion of resolution testing following TC of stools that were negative by the CCNA method but positive with PCR on rectal swabs and/or stool specimens, 8 stool specimens and 10 rectal swabs were PCR positive but CCNA and TC negative (false-positive PCR results). Also, four stool specimens and four rectal swabs were PCR negative and CCNA or TC positive (false-negative results) (Table 3).

### Effect of fecal load on the performance of PCR on swab samples

We explored the association between fecal load and assay performance on swab specimens using visible soiling as a surrogate marker. There was no difference in the positivity rate between soiled and unsoiled swabs by PCR [83/528 (15.7%) vs 14/95 (14.7%), respectively, *P* = 0.81].

## DISCUSSION

The current strategy to detect *C. difficile* (and hence diagnose *C. difficile* infection in symptomatic patients) is based on stool specimens (30). However, stool specimens are cumbersome to collect as patients must wait for the occurrence of a bowel movement to produce it. Also, uncooperative patients may fail to notify nursing staff of bowel movements, and collecting stools from patients with altered mental status and patients who are incontinent is even more difficult, if not impossible for patients with toxic megacolon. Many of these barriers to specimen collection could be overcome using a rectal swab specimen instead of a stool specimen. The use of rectal swabs instead of stool samples was shown to accelerate diagnosis in a prospective study in the U.S., allowing more efficient management. In this study of swabs vs stool samples to diagnose CDI, the time to diagnosis was more than 50% faster using rectal swabs than stool samples (0.5 vs 1.2 days, *P* < 0.001) (22).

In this study, we have evaluated and compared the performance of our new *C. difficile* PCR assay targeting the *tcdB* gene using stool and rectal swab of patients suspected of CDI against a standard reference test (CCNA) performed on stools (10, 12). The CCNA method is considered one of the most sensitive and specific methods for *C. difficile* toxin detection (12). *C. difficile* was detected more often by PCR than by the CCNA method on both stools and rectal swabs. Indeed, 6.3% (35/559) and 6.4% (36/559) of participants with CCNA-negative stool specimens were PCR positive on stool specimens and rectal swabs, respectively. To resolve these discrepant results, toxigenic stool culture, which is another widely used reference method for CDI diagnosis (10, 12), was performed on the stool of participants with CCNA-negative stools and PCR-positive rectal swabs and/or stools. Following toxigenic stool culture for these participants, 1.5% (8/532) and 1.9% (10/532) of participants had CCNA/TC-negative stool specimens and PCR-positive stool specimens and rectal swabs, respectively. A total of 7 of the 10 participants showing a false-positive PCR result with stools and/or rectal swabs following toxigenic stool culture had received antimicrobial agents active against *C. difficile* (vancomycin or metronidazole), which may have reduced the ability to grow. The three other PCR-positive, CCNA/TC-negative specimens may be attributable to the presence of *C. difficile* load that was below the detection limit of both CCNA and TC methods. The targets of these two reference methods differ, and each of these methods has its limitations: CCNA detects the production of toxin B *in vivo,* which depends on the level of toxin production by the *C. difficile* strain present in stool, while TC detects the presence of a *C. difficile* strain that is able to produce toxins *in vitro* and depends on its capacity to grow in the culture media. Testing of samples with enzyme immunoassay, which detects glutamate dehydrogenase, an enzyme that is produced by both toxigenic and non-toxigenic *C. difficile* and which is not dependent on toxin production or growth capacity, could have helped to resolve some of the discrepant results. The higher rate of *C. difficile* detection by PCR compared to CCNA and antigenic methods has been reported by many research groups (26, 31–35), and the use of PCR as a standalone assay for CDI diagnosis remains a controversial issue (11, 12, 36). The reason is that asymptomatic carriers of toxigenic *C. difficile* with diarrhea for other reasons (e.g., laxatives) may be positive by PCR, leading to unnecessary treatment and increasing CDI rates (12, 37, 38). However, underreporting CDI using rapid methods that are more specific but less sensitive is also an important issue (36). Therefore, diagnostic stewardship for the use of PCR is imperative, and there should be established institutional criteria for patient stool submission for *C. difficile* detection (12). Currently, PCR is increasingly used for the diagnosis of CDI, and a recent study showed that it is now used in 83% of laboratories in the U.S. (39).

Our findings demonstrate that the diagnostic performance of our new PCR assay for the detection of *C. difficile* in stool specimens and rectal swabs was excellent and comparable to CCNA. Therefore, our assay can be used for the detection of toxigenic *C. difficile* in symptomatic patients suspected of CDI using both types of specimens. In addition, even though numerous laboratories include the absence of visible soiling on swabs as a rejection criterion (16), our assay performed similarly well regardless of whether the swab was visibly soiled with fecal material or not. Performing the assay on unsoiled swab samples may simplify the collection and decrease the rate of rejection. A growing number of studies have reported the reliability of using rectal swabs with *C. difficile* PCR tests (commercial and in-house) for detecting CDI (21, 22, 24, 25, 40). The effectiveness of PCR on rectal swabs for the detection of other enteric infections (bacterial, viral, and parasitic) has also been demonstrated by several groups (21, 40–43). Reducing delays in specimen collection could considerably reduce the overall turnaround time for the diagnosis of CDI and other enteric infections and improve the management of these infections. It should also provide a useful alternative to better control the spread of *C. difficile* and other enteric pathogens in outbreak management including in outpatient settings and public health.

## SUPPLEMENTAL MATERIAL

Supplemental material spectrum.00225-24-s0001.docx Table S1.

ASM does not own the copyrights to Supplemental Material that may be linked to, or accessed through, an article. The authors have granted ASM a non-exclusive, world-wide license to publish the Supplemental Material files. Please contact the corresponding author directly for reuse.
