# Supplementary material for: Comparison of rectal swabs and fecal samples for the detection of Clostridioides difficile infections with a new in-house PCR assay
Source: Microbiol Spectr. 2024 Apr 30;12(6):e00225-24. doi: 10.1128/spectrum.00225-24 (PMC11237655; doi:10.1128/spectrum.00225-24)
Supplement: Supplemental material — Table S1. [file spectrum.00225-24-s0001.docx]

**Table S1.** Oligonucleotides used in this study

| Target | Forward primer sequence | Reverse primer sequence | Probe sequence ^a^ |
| --- | --- | --- | --- |
| *C. difficile tcdB* | GAAGTAAATACTTTAAATGCTGC GAAGTGAATACTTTAAATGCTGC | CTAATTCAACAACTTTGGCTG CTAATTCAACAACTTTAGCCG | TA**G**TT**C**TAAA**G**AAT**C**T**C**TTAGTA |
| Internal control | CAGGTGAAAATGAAGTAGGC | GATCAAATGACGGAGCTTG | TA**G**TT**C**TAAA**G**AAT**C**T**C**TTAGTA |

^a^ Bold nucleotides represent LNA residues
